# Diversity patterns, *Leishmania* DNA detection, and bloodmeal identification of Phlebotominae sand flies in villages in northern Colombia

**DOI:** 10.1371/journal.pone.0190686

**Published:** 2018-01-10

**Authors:** Camila González, Cielo León, Andrea Paz, Marla López, Gisell Molina, Diana Toro, Mario Ortiz, Juan Manuel Cordovez, María Claudia Atencia, Germán Aguilera, Catalina Tovar

**Affiliations:** 1 Centro de Investigaciones en Microbiología y Parasitología Tropical, CIMPAT, Departamento de Ciencias Biológicas, Universidad de los Andes, Bogotá, Colombia; 2 Grupo de Biologia Matematica, BIOMAC, Departamento de Ingenieria Biomedica. Facultad de Ingenieria, Universidad de Los Andes, Bogotá, Colombia; 3 Facultad de Ciencias de la Salud. Universidad del Sinú, Montería, Córdoba, Colombia; 4 Grupo de Enfermedades Tropicales y Resistencia Bacteriana, Facultad de Ciencias de la Salud. Universidad del Sinú, Montería, Córdoba, Colombia; Centro de Pesquisas René Rachou, BRAZIL

## Abstract

Leishmaniases are neglected tropical diseases exhibiting complex transmission cycles due to the number of parasite species circulating, sand fly species acting as vectors and infected mammals, including humans, which are defined in the New World as accidental hosts. However, current transmission scenarios are changing, and the disease is no longer exclusively related to forested areas but urban transmission foci occur, involving some species of domestic animals as suspected reservoirs. The aim of this study was to determine the transmission cycles in urban environments by evaluating sand fly diversity, detection of *Leishmania* DNA, and bloodmeal sources through intra and peridomestic collections. The study was carried out in Colombia, in 13 municipalities of Cordoba department, implementing a methodology that could be further used for the evaluation of vector-borne diseases in villages or towns. Our sampling design included 24 houses randomly selected in each of 15 villages distributed in 13 municipalities, which were sampled in two seasons in 2015 and 2016. Sand flies were collected using CDC light traps placed in intra and peridomestic habitats. In addition to the morphological identification, molecular identification through DNA barcodes was also performed. A total of 19,743 sand flies were collected and 13,848 of them (10,268 females and 3,580 males) were used in molecular procedures. Circulation of two known parasite species–*Leishmania infantum* and *Leishmania panamensis* was confirmed. Blood source analyses showed that sand flies fed on humans, particularly in the case of the known *L*. *infantum* vector, *P*. *evansi;* further analyses are advised to evaluate the reservoirs involved in parasite transmission. Our sampling design allowed us to evaluate potential transmission cycles on a department scale, by defining suspected vector species, parasite species present in different municipalities and feeding habits.

## Introduction

Leishmaniases belong to the group of neglected tropical diseases affecting mainly low-income populations in Africa, Asia and Latin America. Transmission of the disease is strongly related to socioeconomic factors such as malnutrition and population displacement, among others [1]. Every year, 700,000 to one million new cases are recorded, 50,000 to 90,000 of visceral leishmaniasis (VL), and 20,000 to 30,000 deaths. For the cutaneous form of the disease (CL), the most common, one million cases are reported annualy [1]. A mucocutaneous (MCL) form of the disease has also been described, which develops as a late reaction to previously untreated cutaneous lesions and may affect nasal cavities, the septum and the palate [2,3]. Urbanization of the leishmaniases has been recorded, particularly regarding the visceral form caused by *Leishmania infantum* [4,5,6] as well as variation in species’ spatial distribution and vector-parasite interactions [7,8].

The ecology of leishmaniases transmission is complex, due to the number of parasite species circulating (20 known to infect humans, 10 with public health importance), sand fly species acting as vectors (47 reported as proven in the New World) and mammals infected [7,9–11]. There is a lack of information regarding species acting as reservoirs in transmission foci. Although in America several groups of both wild and domestic mammals (rodents, marsupials, primates, canids, bats, and others) have been found to be infected with the parasites [12], their incrimination as reservoirs requires further research and is hard to accomplish [13,14]

Regarding vector species incrimination, procedures used for species identification such as clearing, make it difficult to perform molecular analyses for parasite detection. Establishment of vector feeding habits through bloodmeal source identification is also difficult because specimens must be processed within 96 hours of feeding to avoid blood digestion [15]. These difficulties impose important challenges from the perspective of disease ecology, since the identification of *Leishmania* parasite species infecting vectors and mammals is a critical step in the definition of transmission cycles, and their threat to humans [16].

In 2015, Colombia was among the ten countries recording the highest prevalence of leishmaniases in the world [1], while 10,743 cases of leishmaniasis were reported in 2016, 98.3% of them were of CL [7]. Leishmaniasis is an endemic disease commonly located in rural areas in the country, but can also be found in urban and peri–urban areas [17,18]. The disease is widely spread in the country, while VL foci are principally identified in the Magdalena River Valley region and the Caribbean coast. One important VL transmission focus is located in the department of Córdoba, in the municipality of San Andrés de Sotavento. In 2015 and 2016, Córdoba had the second highest number of VL cases in the countrywith seven and five cases reported each year respectively. Nevertheless, the greatest number of cases reported in the area corresponds to CL, with 218 cases recorded in 2015 and 105 in 2016. The locality of Tierralta contributed to more than half of these cases, however there is no local transmission in the municipality and all the cases seem to belong to soldiers of the Colombian Army performing surveillance in forested areas [19]. Parasites known to be circulating in the department are *Leishmania braziliensis* and *Leishmania panamensis* [20], while 15 species of phlebotomine with medical importance have been reported: *Migonemyia migonei* [21], *Lutzomyia gomezi* [7,22,23], *Psychodopygus panamensis* [7,22,23], *Pintomyia evansi* [7,24,25], *Micropygomyia cayennensis* [22,23], *Pintomyia rangeliana* [22], *Micropygomyia trinidadensis* [23,25], *Evandromyia dubitans*, *Psathyromyia carpenteri*, *Pressatia camposi*, *Pressatia dysponeta*, *Micropygomyia micropyga*, *Nyssomyia yuilli yuilli*, *Micropygomyia atroclavata* and *Psathyromyia shannoni* [23]. In spite of the high number of cases occurring in the department, it has been described as one of the regions with the lowest sand-fly collection records [7]. The lack of eco-epidemiological studies contributing to the understanding of disease transmission is remarkable, possibly due to the political instability of the region that has always posed a challenge to fieldwork studies.

From this perspective, the aim of this study was to determine the transmission cycles in urban environments by evaluating phlebotomine sand fly diversity, *Leishmania* parasite DNA detection, and bloodmeal sources through intra and peridomestic collections. With this approach, we wish to provide an overview of urban transmission cycles using a sampling methodology for zoonoses and vector borne diseases that could be implemented in eco-epidemiological studies in villages in other regions.

## Materials and methods

### Study site

**Fig 1:**
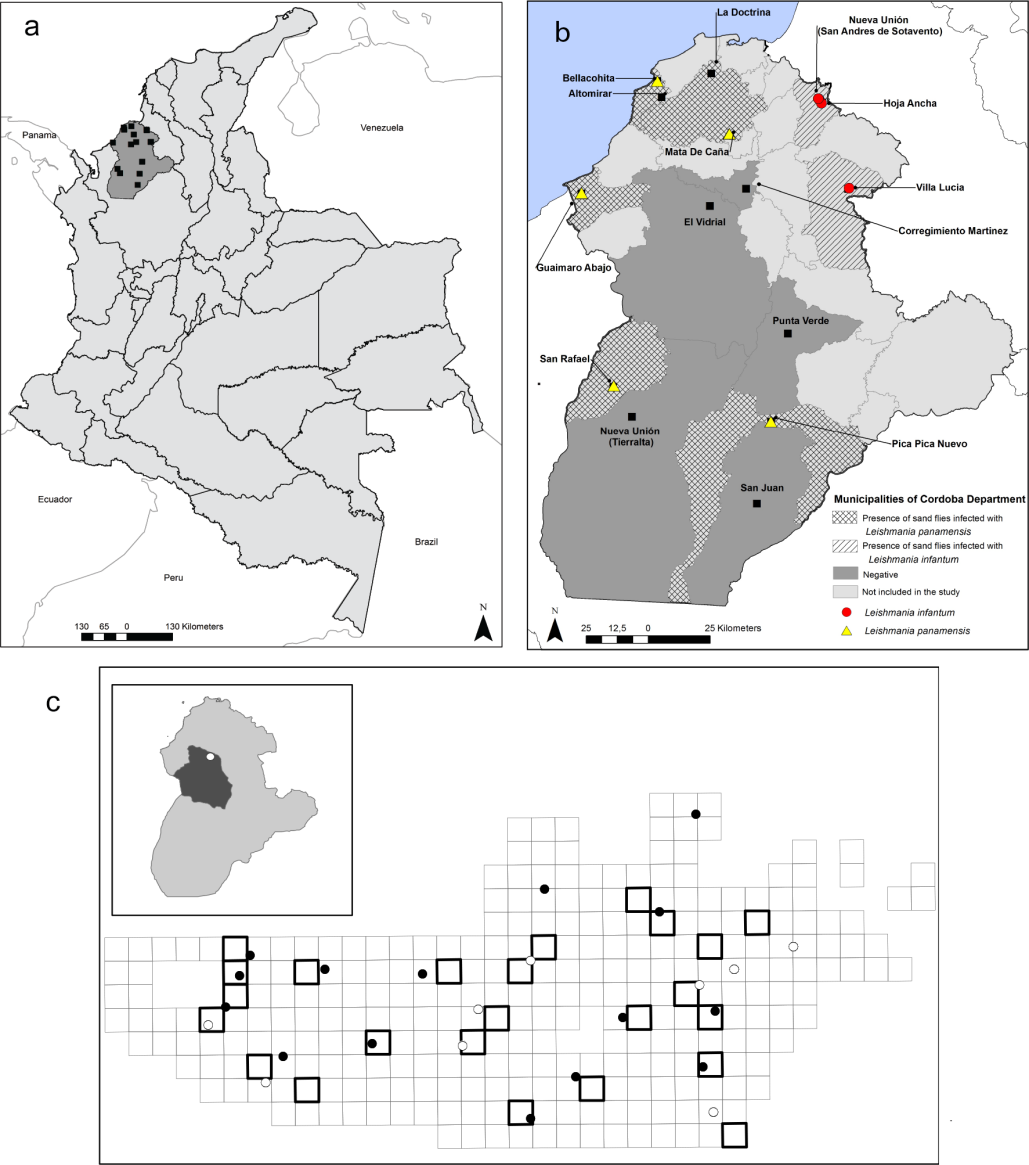
Sampling localities in the Cordoba department in Colombia. a. Córdoba department location in the country. b. Fifteen sampled localities in 13 municipalities of the Cordoba department. Colors correspond to the distribution of *Leishmania* species, in red *Leishmania infantum*, in yellow *L*. *panamensis* and in black localities with no parasites detected. c. Gridded sampling design with a 25m * 25m resolution built using satellite images to identify houses. On the grid 24 random cells were selected and the houses present inside were sampled in each locality. This grid is an example that corresponds to the Vereda el Vidrial, Cordoba, Colombia.

The department of Córdoba, in northwest Colombia, has an elevational range between 260 and 2200 m.a.s.l. Fifteen localities within 13 municipalities out of the 30 present in the department were selected to perform entomological collections, based on accessibility and security (Fig 1, S1 Table). Within each selected locality a total of 24 houses were randomly sampled. In localities for which satellite images with enough resolution to distinguish houses were available in Google Earth [Google Earth (Version 7.1.8.3036) [Software]. Mountain View, CA: Google Inc. (2009). Available from https://earth.google.com/] we spatially randomized house selection (Fig 1). We created a fishnet of 25m*25m covering all the area with houses in ArcMap 10.1 (ESRI, Redlands, CA, USA) and randomly selected 24 cells for sampling (Fig 1). Seven localities did not have available satellite images for sampling design (Villa Lucia-Sahagún, San Juan-Puerto Libertador, Hoja Ancha-San Andrés De Sotavento, Punta Verde-Planeta Rica, Altomirar-Moñitos, Guaimaro abajo-Los Córdobas and Pica Pica-Montelibano) so an initial reconnaissance trip was made to georeference all the houses. Posteriorly, all houses were numbered and 24 houses were randomly selected for sampling using R (R Core Team (2015) R: A language and environment for statistical computing).

To evaluate the transmission cycles in urban environments, we performed adult collections in eight houses each night during three consecutive nights (for a total of 24 houses). In each house a CDC light trap was located intradomiciliary and one in peridomiciliary areas. Traps were activated at 6:00 pm and recovered at 6:00 am (sampling effort:12 hours/ night *per* house) for immediate sample processing; insects were sacrificed by putting the collection jars inside a bag containing triethylamine, and posteriorly sorted for each procedure. Each locality was visited twice and all fieldwork was conducted between August 21^st^ 2015 and October 11^th^ 2016. Once sand flies were separated, they were placed in eppendorf tubes containing 70% ethanol. All the collection tubes were taken for taxonomic identification and molecular processing to the Center for Research in Microbiology and Tropical Parasitology (CIMPAT for its initials in Spanish) at Universidad de Los Andes in Bogotá. Sand flies were sorted in groups using external characteristics; 88% of the males and 6% of the females were then clarified and used for species identification following Young and Duncan [26] and Galati [27], and to establish the reference collection. Once the Phlebotominae fauna present in the collection sites was established, the remaining females were identified following external characteristics such as wing venation patterns, pigmentation, and length of the palpomeres, avoiding the clarification process to optimize molecular procedures. When necessary, females were dissected without clarification to complete species identification based on the spermatheca. The identified females were pooled per house with up to 20 individuals per pool to perform *Leishmania* DNA detection as described below; when only one individual was captured, DNA extraction was performed individually. Females containing blood in their abdomens were kept individually to perform blood source analyses. For all processed females, species confirmation was performed using barcode.

### Parasite detection and blood meal analyses

#### Polymerase chain reaction amplification

To detect sand fly natural infection with *Leishmania* parasites, DNA was extracted from pools with a maximum of 20 females, using ZR Tissue & Insect DNA Miniprep kit (Zymo CA, USA). Conventional PCR was performed with previously described primers specific for *Leishmania* heat-shock protein 70 gene (HSP70) from Hernández et al. [28] and rRNA intergenic region (ITS) from El Tai et al. [29]. The reaction was carried out in a volume of 25 μl using paired primers (10 μM each) and 2x GoTaq Green Master Mix (Promega WI, USA). After an initial denaturation at 95°C for 5 min, PCR amplification was performed with 40 cycles of denaturation (95°C, for 1 min), annealing (60°C, for 1 min), and polymerization (72°C, 1 min), followed by a final extension at 72°C for 10 min. The PCR products were resolved by agarose gel electrophoresis, generating a product with a length of 300–350 bp.

#### High Resolution Melting HRM

Real-time PCR was coupled with HRM analysis using the HSP70 and ITS1 genes in a Real-time PCR system 7500 (Applied Biosystems, Inc., CA, USA) with 21 μL amplification reactions. The reaction mix contained 1X of Master Mix MeltDoctor HRM (Applied Biosystems, Inc., CA, USA), a 5 μM solution of each primer HSP70F (5’ AGG TGA AGG CGA CGA ACG 3’) and HSP70R (5’ CGC TTG TCC ATC TTYGCG TC 3’) (with one variation in one nitrogenous base) for the amplification of HSP70 and for ITS1, LITSR (5’ CTG GAT CAT TTT CCG ATG 3’) and L5.8S (5’ TGA TAC CAC TTA TCG CAC TT 3’) and 10 ng/μL of DNA template. Real-time PCR cycle conditions were adjusted to the protocol described by Hernadez et al [28] with variation in denaturation time (95°C during 30 sec) and High Resolution Melting at 95°C for 45 seconds.

#### Blood source analysis

To analyze the feeding preferences in each environment (intra and peridomicile), blood-fed females were stored in individual eppendorfs with 70% ethanol. Blood source identification was carried out using cytochrome b sequences. All DNA templates were tested with two primer pairs, *mammals C* described by Molaei et al [30] and a primer pair for avian species (GCCAAATATCATTCTGAGGGGC[f], GGCGAATAGAAAATATCATTGTGG[r], 410 bp) described by Ferro et al [7]. Controls were used during extraction and amplification; the negative control for amplification consisted of a pool of sand fly legs, and the positive was DNA from dogs and humans. The amplification products were purified, sequenced and identified by comparing the DNA sequence to GenBank databases using Blast (National Center for Biotechnology Information).

#### DNA barcoding

To confirm species identification, a subsample of the collected specimens was DNA barcoded. The DNA barcode region from the COI gene was PCR-amplified from individual sand flies of both sexes. DNA extracts were prepared from a small sample of leg tissue or abdomen tissue in individuals lacking appendages and extraction was performed using the ZR Tissue & Insect DNA Miniprep kit (Zymo CA, USA). A PCR reaction, 1 μl total DNA were mixed with the following reagents: 12.5 μl of 2x GoTaq Green Master Mix, 10 μM of forward and reverse primers LCO1490 (5’- GGTCAACAAATCATAAAGATATTGG-3’) and HCO2198 (5’- TAAACTTCAGGGTGACCAAAAATCA-3’) [31] to a final reaction volume of 25μl. The thermo cycling conditions consisted of one cycle of 1 min at 94°C, 40 cycles of 40 s at 94°C, 40 s at 52°C, and 1 min at 72°C, and finally 5 min at 72°C, the size of the specific PCR product was 658 bp. All sand fly individuals that were *Leishmania* positive through HRM were confirmed by sequencing of the parasite CytB, and therefore only the infected specimens with an elevated identification percentage (97% or higher) in Blast were accepted as positive [20].

### Eco-epidemiological and spatial analyses

#### Spatial and temporal distribution

To evaluate patterns of species’ spatial distribution, we tested for spatial aggregation of all vector species, *P*. *evansi* alone and all parasites separately using Moran’s I index for spatial autocorrelation in ArcMap 10.2 [ESRI 2011. ArcGIS Desktop: Release 10.2. Redlands, CA: Environmental Systems Research Institute]. Community analyses for sand-fly composition were done using the vegan package for R [R Core Team (2017). R: A language and environment for statistical computing. R Foundation for Statistical Computing, Vienna, Austria. URL https://www.R-project.org/], we computed the Bray-Curtis dissimilarity between sites and a posterior cluster analysis for visualization as implemented in the vegan package for R [R Core Team (2017). R: A language and environment for statistical computing. R Foundation for Statistical Computing, Vienna, Austria. URL https://www.R-project.org/]. The same analysis was repeated for peridomiciliary and intradomicilary samples separately and we computed the average distance between the two sampled communities using the Bray-Curtis and Jaccard indexes.

#### Epidemiological information

To evaluate infestation rates per house and identify infection patterns, a household survey designed for this study was performed. For each sampled house, we recorded the number of inhabitants, the presence of animals (divided in 11 categories) and the use of insecticides. We measured the correlation between the abundance of sand flies and the number of inhabitants and animals with a spearman correlation test. We also evaluated the relation between the use of insecticides and the abundance of insects. Analyses were performed separately for *L*. *infantum* and *L*. *panamensis* vectors.

Additionally, the relation between presence of parasite in sand flies and occurrence of human cases in the municipality was assessed. To establish infection rates since results were obtained by pool, we calculated the minimum infection rate assuming at least one infected sand fly per positive pool. The number of cases was recorded by the National System of Public Health Vigilance (SIVIGILA by its Spanish acronym) and were obtained for each municipality during the time of the study. All statistical analyses were done in R [R Core Team (2017). R: A language and environment for statistical computing. R Foundation for Statistical Computing, Vienna, Austria. URL https://www.R-project.org/].

## Results

### Adult collection and species identification

A total of 19,743 phlebotomine sand flies were captured (15,527 females and 4,216 males), and 70% of the collected individuals (10,268 females and 3,580 males) were processed for species identification and molecular analyses. The remaining individuals belonged to a single municipality, San Andrés de Sotavento (5,945 individuals) and to a single species, *P*. *evansi*, but were not processed due to time limitation (Table 1).

**Table 1:** Total number of collected sand flies by species in each of 15 localities in the Cordoba department in Colombia. Numbers correspond to number of captured individuals for both intra and peridomicilairy traps of 24 houses in each village. Village code: CGA: Los Cordobas-Guaimaro Abajo, TNU: Tierra Alta-Nueva Union; Sahagun-Villa Lucia; LMC: Lorica- Mata de Caña, MPP: Montelibano- Pica Pica Nuevo, MV: Monteria-El Vidrial, PLSJ: Puerto Libertador-San Juan, LD:Lorica-La Doctrina; MAL: Moñitos-Altomirar.

Sand fly Species/ Village code	CGA	CM	LD	LMC	MAL	MBE	MPP	MV	PL	PV	SHA	SNU	SVL	TNU	VSR	TOTAL	Percent
*E*. *dubitans*	77		2	-	14	50	13	-	-	6	96	158	-	3	6	425	3.07%
*M*. *cayennensis*	96	7	4	2	228	162	165	18	10	111	99	107	192	27	21	1249	9.02%
*L*. gomezi*	459	4	1	59	61	88	29	3	42	15	55	64	27	60	16	983	7.10%
*M*. *trinidadensis*	4		-	-	13	16	15	-	3	-	1	12	6	1	13	84	0.61%
*P*. evansi*	3		-	1	407	593	-	-	-	32	4156	4360	573	1	2	10128	73.14%
*P*. *rangeliana*	18	2	3	3	25	43	11	1	1	9	6	43	33	1	1	200	1.44%
*P*. *camposi*	-		-	-	-	-	-	-	1	-	-	-	-		-	1	0.01%
*P*. *dysponeta*	-		-	-	-	-	35	-	-	-	-	-	-	3	6	44	0.32%
*P*. *carpenteri*	33		-	-	49	-	15	-	1	2	-	-	-	4	-	104	0.75%
*P*. panamensis*	19		-	1	3	29	20	-	59	7	52	75	1	136	226	628	4.53%
*P*. shannoni*	1		-	-	-	-	-	-	-	-	-	1	-	-	-	2	0.01%
**TOTAL**	710	13	10	66	800	980	303	22	117	182	4465	4820	839	236	291	13848	
** Percent**	5.13%	0.09%	0.07%	0.48%	5.78%	7.08%	2.19%	0.16%	0.84%	1.31%	32.24%	34.81%	6.06%	1.70%	2.10%		

Species known as proven or suspected vectors.

Species morphologic identification following the clarification process was performed in 6% of the collected females (n = 618), and 90% of the males (n = 3,140). Non-clarified females were identified using external characters as described in the methods section, and 20% of them were dissected for confirmation using the spermatheca. All the non-clarified females were processed for molecular analyses. The confirmation of morphological identification was performed in 101 individuals using DNA barcoding, excluding blood fed females (Table 2). In total, eleven species were identified; *Pintomyia evansi* was the most abundant species (71% of all the identified sand flies) followed by *Micropygomyia cayennensis* 9.01% and *Lutzomyia gomezi* 7.09%.

**Table 2:** Results from DNA barcode analyses for species identification confirmation. Samples were collected in 24 houses in each of 15 localities in the Cordoba department in Colombia a subset of the collected specimens were DNA barcoded suing standard protocols and primers. The table shows the species identity according to morphology-based taxonomy and the reference sequence genbank number and ID for that species. All the samples belonged to single female specimens except for *P*. *evansi* specimens that were a pool of 10 individuals. We show the percentage of barcodes produced for each nominal species that matched the reference sequence, in parenthesis the number of matching nucleotids and total number of produced barcodes. References correspond to publications of the reference sequences.

Species determination by taxonomy	GenBank Access number	Query cover	% Identity	Species	Reference
*Psychodopygus panamensis*	GU909460.1	99%	98% (669/683)	*Lutzomyia panamensi*	**[32]**
*Micropygomyia cayennensis*	GU909472.1	96%	99% (679/682)	*Lutzomyia cayennensis cayennensis*	**[32]**
*Pintomyia evansi*	GU909458.1	100%	98% (659/662)	*Lutzomyia evansi*	**[32]**
*Lutzomyia gomezi*	KC921248.1	98%	98% (685/699)	*Lutzomyia gomezi*	**[33]**
*Lutzomyia shannoni*	GU909469.1	95%	98% (234/238)	*Lutzomyia shannoni*	**[32]**
*Micropygomyia trinidadensis*	GU909498.1	97%	100% (658/681)	*Lutzomyia trinidadensis*	**[32]**
*Pintomyia rangeliana*	GU909493.1	98%	99%(675/681)	*Lutzomyia rangeliana*	**[32]**
*Evandromyia dubitans*	GU909446	97%	99% (677/682)	*Lutzomyia dubitans*	**[32]**
*Psathyromyia carpenteri*	GU909444.1	99%	98% (668/683)	*Lutzomyia carpenteri*	**[32]**
*Presatia dysponeta*	GU001732.1	71%	97% (302/314)	*Lutzomyia dysponeta*	**[34]**

Regarding species richness, at the scale of sampling localities, *M*. *cayennensis*, *P*. *rangeliana* and *L*. *gomezi* were collected in the 15 sampled localities, while *P*. *camposi*, was the rarest species, with only one individual present in San Juan (Puerto Libertador).

**Fig 2:**
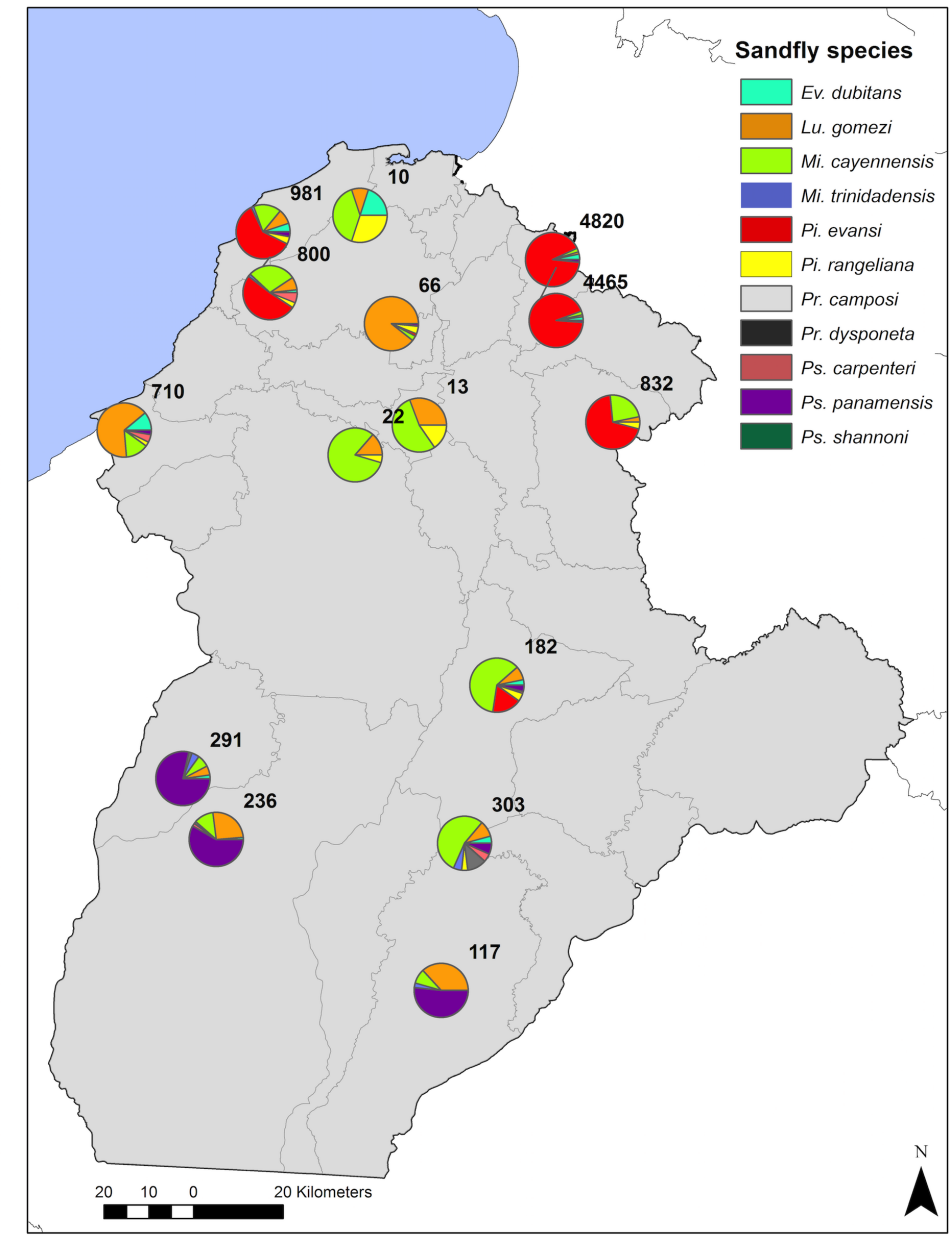
Sand fly species diversity and community composition in each of the 15 sampled localities in the Cordoba department in Colombia. Diversity was computed as total number of sand flies captured by locality (grouped from 24 houses, sampled both intra and peridomiciliary). Different colors represent different species of sand flies identified with traditional morphological techniques and confirmed via DNA barcoding.

Nueva Unión, Los Córdobas and Tierralta were the sites with the greatest species richness, since nine sand fly species were present out of the eleven identified. The localities of Martinez and El Vidrial, on the other hand, had only three species of sandlies. Abundances in La Doctrina and Martinez were very low with a total of 13 and 10 sand flies for each site, while the localities with the highest number of captured sand flies were Nueva Unión (n = 4,820) and Hoja Ancha (n = 4,465), which are both located in the township of San Andres de Sotavento. Summing the latest two localities *P*. *evansi* accounted for more than 80% of the species presence in the whole department. Furthermore, this species was collected in every sampled house in ten localities. In Nueva Unión, a single house had 10% of the collections made for this species in the whole study (1, 048 individuals, 784 of them collected intradomiciliary) (Fig 2).

In general, 68% of the collected specimens were found peridomiciliary and 31.2% intradomiciliary, however, *M*. *cayennensis* was predominantly collected intradomiciliary and *P*. *shannoni* was collected in equal proportions in traps placed inside and outside of the households. Regarding localities, Punta Verde was the only one where more insects were collected inside households (53.3%), and equal proportions were obtained for El Vidrial.

### Parasite detection and blood meal analyses

From the total sample of identified females, 90% (9,293) were processed to detect infection, and to identify the parasite species involved. Infection was analyzed by location, taking into account the number of houses where phlebotomine sand flies were identified as positive for *Leishmania* parasites (S2 Table).

After sequencing positive samples, two parasite species *Leishmania infantum* and *Leishmania panamensis* were identified in 24 pools containing from a single sand fly up to 20. *Leishmania infantum* was found in 16 pools from five households in three localities: Nueva Unión (three households, 13 positive pools) and Hoja Ancha (one household, two positive pools) in San Andres de Sotavento, and Villa Lucia in Sahagun (one household, one positive pool). Species identified as suspected vectors for the transmission of *Leishmania panamensis* based on their natural infection were *L*. *gomezi*, *M*. *cayennensis* and *P*. *panamensis*. Seven houses in five localities had infected sand flies with *Leishmania panamensis*. In tree localities (Moñitos, Los Cordobas and Lorica), only *L*. *gomezi* was found infected, while one pool of 11 *M*. *cayennensis* was found infected in Montelibano, and one pool of 16 *P*. *panamensis* females was found naturally infected in Valencia (Fig 1, Table 3). Regarding the minimum infection rate, the lower were obtained for *P*. *evansi* in Hoja Ancha and Villa Lucia and for *L*. *gomezi* in Moñitos. The highest infection rates were obtained for *L*. *gomezi* in Lorica because only 16 individuals were collected.

**Table 3:** Infection results of screened pools. For each sampled village the minimum infection rate (MIR,) the parasite species detected and the infected sand fly species are shown.

Municipality	Village	Total screened	Positive pools	Minimum infection rate ^a^	Sand fly species	Parasite species
Lorica	Mata de caña	16	1 pool (1 individual)	6.25	*L*. *gomezi*	*Leishmania panamensis*
Sahagún	Villa Lucía	450	1 pool (7 individuals)	0.22	*P*. *evansi*	*Leishmania infantum*
San Andres de Sotavento	Nueva Unión	3120	9 pools (10 individuals) 4 pools (20 individuals)	0.42	*P*. *evansi*	*Leishmania infantum*
Moñitos	Bellacohita	655	1 pool (7 individuals)	0.15	*L*. *gomezi*	*Leishmania panamensis*
Los Córdobas	Guaimaro Abajo	405	2 pool (20 individuals) 1 pool (6 individuals)	0.74	*L*. *gomezi*	*Leishmania panamensis*
Montelíbano	Pica Pica Nuevo	187	1 pool (11 individuals)	0.53	*M*. *cayennensis*	*Leishmania panamensis*
Valencia	San Rafael	214	1 pool (16 individuals)	0.47	*P*. *panamensis*	*Leishmania panamensis*
San Andres de Sotavento	Hoja Ancha	3514	1 pool (20 individuals) 1 pool (18 individuals)	0.06	*P*. *evansi*	*Leishmania infantum*

^a^ Minimum infection rate = infected sand flies *100/total number of captured sand flies. Assuming at least one infected sand fly by positive pool.

### Blood source analysis

In total, 50 fed females were found and blood source species identification was performed. Half of the processed samples were identified as *P*. *evansi* (n = 26), followed by *L*. *gomezi* (n = 9), *P*. *panamensis* (n = 7), *P*. *rangeliana* (n = 4), *M*. *cayennensis* (n = 3) and *M*. *trinidadensis* (n = 1). Species confirmation was performed trough barcode, and no misidentifications were found (Table 4). To further confirm the absence of contamination with human DNA, we performed an additional experiment amplifying a 268bp fragment of the human beta globin gene (primers GH20/PC04) on 35 original sand flies DNA extracts. We managed to obtain amplification in 80% of the samples, confirming the presence of human DNA before any procedure. The remaining 20% (7 individuals) could be either degraded or contaminated during the process.

**Table 4:** Blood sources detected in sand fly specimens in each locality. Blood fed females were processed individually, identified based on external characters, and species confirmation was performed using DNA barcode procedures.

Municipality	Total screened	Morphological species identification	Species confirmation using barcodes	GenBank Access number	Blood source
	(individually)				
Cereté	1	*L*. *gomezi*	*L*. *gomezi*	KC921254.1	*Gallus gallus*
Lorica	1	*L*. *gomezi*	*L*. *gomezi*	KC921254.1	*Canis lupus familiaris*
	1	*M*. *cayennensis*	No amplification		*Canis lupus familiaris*
	1	*M*. *cayennensis*	No amplification		*Anas platyrhynchos domesticus*
	1	*P*. *evansi*	*P*. *evansi*	GU909458.1	*Homo sapiens*
	1	*P*. *rangeliana*	No amplification		*Homo sapiens*
Los Córdobas	1	*L*. *gomezi*	*L*. *gomezi*	KC921254.1	*Homo sapiens*
	1	*P*. *panamensis*	*P*. *panamensis*	GU909460.1	*Marmosa robinsoni*
Montelíbano	1	*L*. *gomezi*	*L*. *gomezi*	KC921254.1	*Homo sapiens*
Montería	1	*M*. *cayennensis*	No amplification		*Homo sapiens*
Moñitos	1	*L*. *gomezi*	No amplification	KC921247.1	*Gallus gallus*
	1	*L*. *gomezi*	*L*. *gomezi*	KC921254.1	*Homo sapiens*
	1	*L*. *gomezi*	No amplification	KC921254.1	*Sus scrofa domestica*
	1	*P*. *evansi*	*P*. *evansi*	GU909457.1	*Homo sapiens*
	1	*P*. *rangeliana*	No amplification		*Homo sapiens*
Planeta Rica	1	*L*. *gomezi*	*L*. *gomezi*	KC921254.1	*Homo sapiens*
	1	*P*. *evansi*	*P*. *evansi*	GU909457.1	*Homo sapiens*
	1	*P*. *rangeliana*	No amplification		*Gallus gallus*
Puerto Libertador	1	*P*. *panamensis*	*P*. *panamensis*	GU909460.1	*Homo sapiens*
Sahagún	1	*P*. *evansi*	*P*. *evansi*	GU909458.1	*Homo sapiens*
	1	*P*. *evansi*	*P*. *evansi*	GU909458.1	*Gallus gallus*
	1	*P*. *rangeliana*	No amplification		*Canis lupus familiaris*
	1	*M*. *trinidadensis*	No amplification		*Cantorchilus leucotis*
San Andres de Sotavento	2	*L*. *gomezi*	*L*. *gomezi*	KC921247.1	*Homo sapiens*
	1	*P*. *evansi*	*P*. *evansi*	GU909458.1	*Canis lupus familiaris*
	18	*P*. *evansi*	*P*. *evansi*	GU909458.1	*Homo sapiens*
	1	*P*. *evansi*	No amplification		*Gallus gallus*
	1	*P*. *panamensis*	*P*. *panamensis*	GU909460.1	*Homo sapiens*
	1	*P*. *panamensis*	*P*. *panamensis*	GU909460.1	*Gallus gallus*
Tierra Alta	1	*P*. *panamensis*	*P*. *panamensis*	GU909460.1	*Homo sapiens*
Valencia	2	*P*. *panamensis*	*P*. *panamensis*	GU909460.1	*Homo sapiens*

The most common blood source was human (72%), followed by chicken (12%) and dog (8%). Other blood sources were present in one specimen of *P*. *evansi* from Altomirar, which fed on pig, and two species feeding on birds: *M*. *cayennensis* on ducks and *M*. *trinidadensis* on *Cantorchilus leucotis*. The only specimen found to be feeding on wild mammals was one *P*. *panamensis* fed on *Marmosa robinsoni*.

*Pintomyia evansi* was the species with highest number of blood fed females and was found feeding on humans, chickens, dogs and pigs. In San Andres de Sotavento, *P*. *evansi* predominantly fed on humans (90% of the samples), one specimen in Hoja Ancha fed on *Gallus gallus* and one in Nueva Unión fed on dog (Table 4).

In other localities where sand flies infected with *L*. *panamensis* were detected and blood-fed females were captured, all the specimens were positive for human blood source: two *L*. *gomezi*, one in Bellacohita and one in Guaimaro Abajo, and two *P*. *panamensis* in San Rafael.

## Eco-epidemiological and spatial analyses

### Spatial and temporal distribution

Considering sand fly spatial distributions, tests of spatial aggregation showed that insects are randomly distributed in the sampling areas (Moran’s I = -0.066, Z-score = -0.32 and P-value = 0.75), a conclusion that holds when studying *P*. *evans*i only (Moran’s I = -0.122, Z-score = -1.11 and P-value = 0.27). The distribution of vectors infected with parasites is also random at the department scale (Moran’s I = -0.076, Z-score = -0.45 and P-value = 0.65).

**Fig 3:**
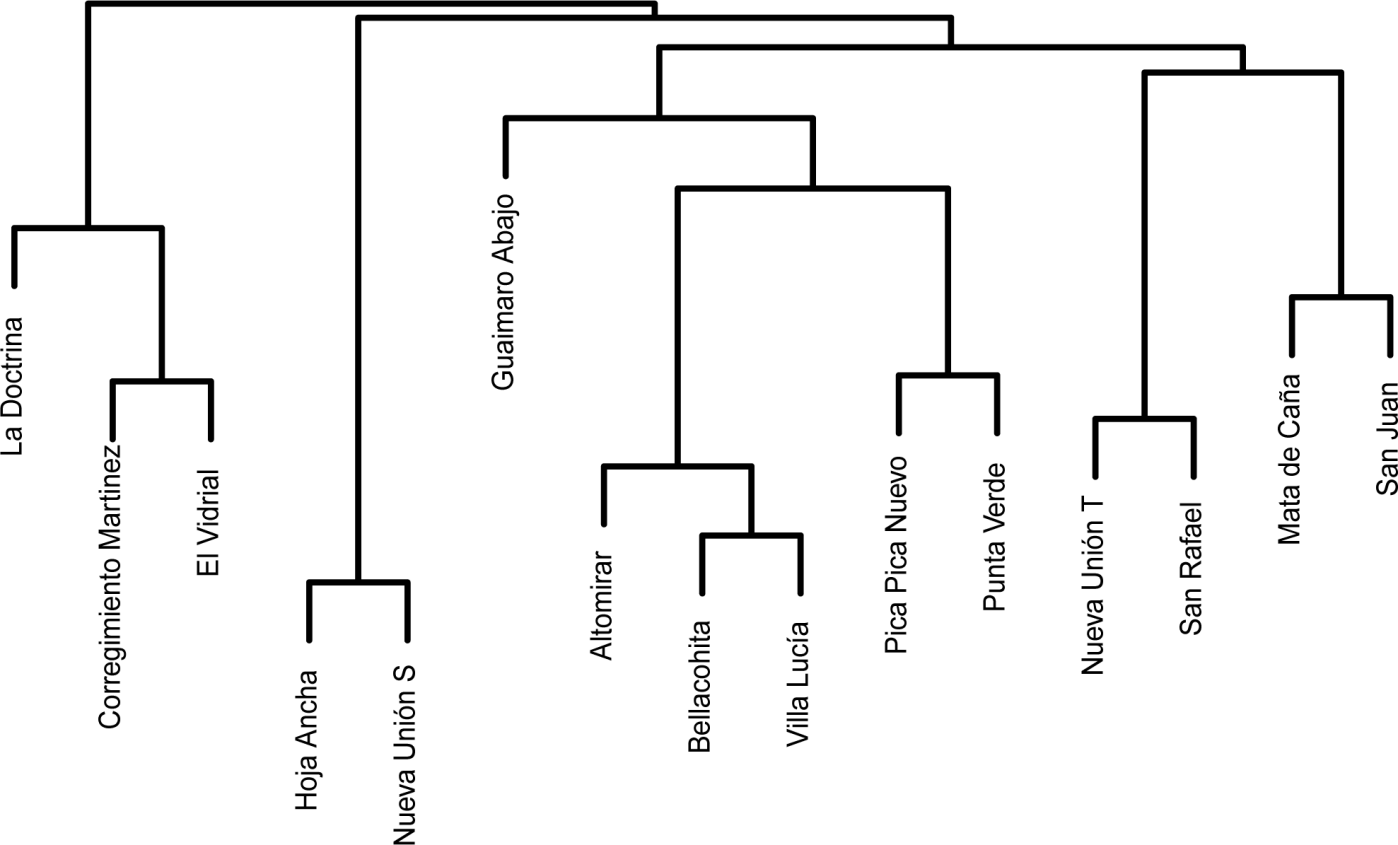
Cluster dendrogram showing similarity of sand-fly community composition of the sampled towns in Cordoba, Colombia. The dendrogram is based on a Bray-Curtis distance with towns clustered together having communities more similar to each other than to other towns. For this dendrogram, sandflies from all houses in a single sampled town were grouped irrespective of wether they were sampled intra or peridomicilary.

Community similarity measures based on sand fly composition showed that towns are not clustered geographically (Fig 3). For example, Bellacohita is more closely related to Villa Lucia than to Altomirar although it is only a few kilometers away from the last one. Another example is that of Mata de Caña (in the north of the Department) and San Juan, the southernmost site sampled, that cluster together according to species composition. That is, presence of certain community assemblages might be driven by other factors such as land cover in the particular site.

Furthermore, community composition is different between intra and peridomicilary samples with them sharing around half of the vector species (Bray Curtis = 0.4209348, Jaccard = 0.592475). When measuring community similarity based on either intradomiciliary samples or peridomicilary samples alone, results in slightly different clustering are obtained. Two sites, Mata de Caña and Guaimaro Abajo have particularly unstable relations when changing the studied community (between intra and peridomiciliary communities). Regarding spatial distribution of infected houses, there was no spatial aggregation or any other identifiable pattern that could explain their distribution in each locality (Fig 3).

### Epidemiological information

Regarding the number of cases recorded in Cordoba during the time of the study, 194 cases from 17 known municipalities were notified to the SIVIGILA. Seven of the VL, three of MCL and 184 of CL. Six of the municipalities that recorded cases were not included in our study and five were positive for sand fly presence but negative for parasite infection. The remaining six municipalities where cases were reported also had records of infected sanflies. San Andrés de Sotavento recorded 12 CL cases and two VL cases, however we detected only *L*. *infantum* present in sand flies. On the contrary, Sahagun didn’t record human cases but one positive pool was detected for *L*. *infantum*; in Moñitos one VL case was recorded but only *L*. *panam*ensis was isolated from sand flies. Regarding the cutaneous and mucocutaneos forms, Lorica reported one MCL case and in this municipality 2/24 households were positive for *L*. *panamensis* in *L*. *gomezi*. Los Córdobas recorded one CL case and we detected 2 /24 households positive for *L*. *panamensis* in *L*. *gomezi*. Lastly, Valencia recorded seven CL cases and *L*. *panamensis* was detected in *P*. *panamensis*, while in Montelibano 4 CL cases were reported and *M*. *cayennensis* was found infected with *L*. *panamensis*.

In general, there was no correlation between the number of infected pools and the local abundances of *P*. *evansi* collected; however, in Nueva Unión, the single household that accounted for 10% of the collected *P*. *evansi* in the whole study, had ten pools positive for *L*. *infantum*. As reported for *L*. *infantum* infection, no correlation was found between the number of infected pools and sand fly local abundances.

The abundance of vectors for both parasites *L*. *infantum* and *L*. *panamensi*, was moderately correlated with the number of animal categories present in the household (ρ = 0.257, p-value = 8.2 x 10^−7^ and ρ = 0.20, p-value = 0.00015 respectively). There was no significant correlation between abundance of vectors for either parasite and inhabitants of the household (ρ = 0.076, *p*-value = 0.152 and ρ = 0.087, *p-*value = 0.149 respectively). No relation was found between insecticide use and the abundance of vectors (p-value = 0.604 and p-value = 0.381 respectively).

## Discussion

In this study, we identified key factors related to *Leishmania* transmission in urban localities: by using different approaches and an important sampling effort we were able to collect and identify phlebotomine sand flies, while also detecting parasite species and blood sources in an accurate way. Our study design can be applied to other areas where the disease exhibits similar occurrence patterns and the acquisition of eco epidemiological information is needed.

Two parasite species were identified: *L*. *infantum*, present in three villages in two municipalities, all located in the northeast, and *L*. *panamensis*, known to cause MCL and CL, in five municipalities, with sparse distribution. However, infection rates in sand flies didn’t reflect the recorded occurrence of cases in the sampled municipalities; possibly infected sand flies are not reaching the threshold necessary to produce human cases, or underreporting of cases from infected localities are missing in the national epidemiological reports. However, we could identify infected sand flies and human blood sources in municipalities where transmission is occurring, providing important information to health authorities, as we will discuss further.

The use of DNA barcodes allowed the confirmation of morphologic identifications for 101 sand fly individuals and flagged only four individuals as misidentifications, reinforcing the reliability of our results. In all cases, the identity of the generated barcodes with the reference database was higher that 97%. In this way, the technique constitutes an important tool to process simultaneously samples both for species identification and parasite detection without compromising the DNA integrity in the clearing process.

Parasite detection and species identification from sand flies was successful in this study, however the HRM technique, with parasite DNA extracted from sand flies did not perform as expected [28]. Therefore, sequencing of the parasite’s Cytb was the best alternative to perform adequate species identification. Although HRM is promising, it needs to be further validated with field samples to achieve a consensus in the algorithm used for species discrimination and the melting temperature ranges for each *Leishmania* species.

All the sand fly species that we found infected have been previously reported in the country as species with medical importance, they have anthropophilic habits, and are widely distributed, characteristics that reinforce their potential to act as vectors [23]. *Micropygomyia cayennensis* is a species widely distributed in Central and South America and has been found infected with unidentified promastigotes in Cordoba, Tolima and Cundinamarca [35]; this species is commonly collected with the primary vectors *P*. *evansi* or *L*. *longipalpis* in VL transmission foci [36,37]. It is known to feed on cold-blooded vertebrates [26] but has also been collected inside households [21] and in high abundances, being the second most abundant species after *P*. *evansi* in Montes de María [26,38]. In high densities, it has been reported biting humans [35]. Regarding *P*. *panamensis* and *L*. *gomezi*, they are known vectors of *Leishmania panamensis* in Panamá [39] and *Leishmania braziliensis* in Venezuela [40,41] and in Colombia both species have been found infected with *L*. *panamensis* in the Boyacá Department [42]. In Colombia, *L*. *gomezi* is distributed in different ecosystems, from moist and dry forest [37, 43, 44] to urban areas where it is known to be highly anthropophilic [45,46]. The importance of this species as a vector is increasing due to its ability to adapt to transformed ecosystems.

Infection rates detected in our study, varied for *L*. *infantum* from 0.06% in Hoja Ancha to 0.42 in Nueva Unión, both located in San Andrés de Sotavento. In the same municipality, Montoya-Lerma et al [47], established *P*. *evansi* infection rates with *L*. *infantum* in 0.05%, while in Venezuela it was 0.23% [47].

Regarding *P*. *panamensis*, infection rates varied from 0.15% to 0.74% in those localities where more than 100 sand flies were processed, and reached 6.25% in Lorica where only 16 individuals were analyzed. In a well-characterized transmission focus in Chaparral, infection rates of *Pintomyia longiflocosa* infected with *L*. *guyanens*is were 0.2% [8]. Insect’s local abundances were not related to infection rates; it is necessary to perform further analysis aiming to define the key parameters involved in parasite presence or prevalence, for example infection of vertebrate species present in households.

The fact that insects were collected in similar proportions inside and outside households suggest that insects attracted to light can easily encounter humans. Although they can be breeding outside, their bloodmeals can occur inside, as the high proportion of human blood source detected in our samples suggests. All the infected sand fly species fed mostly on humans except for *M*. *cayennensis* that was found equally feeding on humans, dogs and birds.

*Pintomyia evansi*, the most abundant species, has been incriminated as the vector of *L*. *infantum* in San Andrés de Sotavento, and dogs and *Didelphis marsupialis* have been identified as the zoonotic reservoirs [48, 49]. A study developed by Montoya-Lerma et al [48], aiming to confirm *P*. *evansi*´s anthropophilic behavior in San Andrés de Sotavento, demonstrated a clear preference of *P*. *evansi* towards human blood over dogs and opossums, and no significant difference in preference between the last two. Nevertheless, preference distinction depended on sand fly abundances; when low densities where identified no significant host preferences were determined but when high density was identified there was a marked preference for humans; in the present study, human blood source was detected in most of the *P*. *evansi* sand flies in localities with overwhelmingly high abundances [48]. It would be important to follow the appearance of cases in these localities since perhaps this scenario can be used as an alert to predict an outbreak. High local abundances of vector species can push a switch in feeding habits increasing the contact between competent vectors and humans; the role of humans as reservoirs has not been established yet, however, in Colombia, there is evidence of *Leishmania* being present in human skin samples in the absence of lesions [50].

A study developed in Sucre, found that dogs are less attractive to sand flies in the presence of other animals like cattle, pigs and donkeys [51]. Pigs and chickens raised close to households constitute other species with epidemiological relevance, since their habitat provides breeding sources for sand flies in peridomestic habitats [52–54]. Birds are refractory to *Leishmania* transmission, but are an attractive blood source for sand flies. Here we detected domestic (chickens and ducks), but also sylvatic (*Thryothorus leucotis*) avian blood sources. On the other hand, avian blood sources could promote a dilution effect, making it likely that in areas where sand flies feed on them, parasite prevalence in insects and mammals, including humans, is low [55]. Nevertheless a study developed by Morrison et al [56] demonstrated a clear preference of *L*. *longipalpis* towards cows and pigs over chickens, and hen houses can actullay be playing the role as attractants to various other reservoirs, including dogs used as protectors, therefore enabling *Leishmania* cycle development [11]. The number of engorged sand flies that we could detect is not enough as to accomplish further analysis on this direction; however the evidence they provide is important and poses important questions to be further addressed. Future work is required to evaluate which could be the main reservoir in the region.

Considering spatial distribution analyses, infected houses do not show spatial patterns of distribution and infection is not correlated to the number of sand flies present in each house either. Studies on breeding sites could contribute exceptional information in order to detect the key factors influencing sand fly local abundances.

From the epidemiological perspective, our sampling design cannot be correlated with the occurrence of cases in the department. Sampling at a local scale can provide useful information, however in order to be able to describe a proper transmission scenario, this kind of study should be established in localities were human cases have been identified so that the link between all the variables and the risk of parasite transmission to humans can be determined.

Although we are detecting natural infection and blood sources, our results are not sufficient to determine variables leading to differential sand fly local abundances and infection. Further analyses are required to better understand how these variables relate to human cases and which species could be acting as reservoirs for the disease.

## Supporting information

S1 Table Localities included in the study. (DOC)

S2 Table Comparison of identification methods for *Leishmania* species. (DOC)
