# Supplementary material for: Diversity patterns, Leishmania DNA detection, and bloodmeal identification of Phlebotominae sand flies in villages in northern Colombia
Source: PLoS One. 2018 Jan 10;13(1):e0190686. doi: 10.1371/journal.pone.0190686 (PMC5761875; doi:10.1371/journal.pone.0190686)
Supplement: S1 Table — (DOC) [file pone.0190686.s001.doc]

| **Municipality** | **Locality** | **Coordinates** | | **Sampling periods (three nights each)** | |
| --- | --- | --- | --- | --- | --- |
|  |  | **(of the house #12)** | |  |  |
|  |  | **Longitude** | **Latitude** | **First** | **Second** |
| Lorica | La Doctrina | -75.89 | 9.293 | 23/08/15 | 9/06/16 |
|  | Mata de Caña | -75.827 | 9.075 | 25/09/15 | 2/09/16 |
| Montería | El Vidrial | -75.9 | 8.811 | 2/10/15 | 7/04/16 |
| Sahagún | Villa Lucía | -75.382 | 8.877 | 8/10/15 | 10/08/16 |
| Cereté | Corregimiento Martínez | -75.771 | 8.871 | 16/10/15 | 21/04/16 |
| Planeta Rica | Punta Verde | -75.615 | 8.348 | 27/11/15 | 13/07/16 |
| San Andrés de Sotavento | Nueva Unión | -75.503 | 9.204 | 16/12/15 | 22/09/16 |
|  | Hoja Ancha | -75.496 | 9.188 | 8/07/16 | 12/10/16 |
| Moñitos | Bellacohita | -76.085 | 9.26 | 19/02/16 | 25/07/16 |
|  | Altomirar | -76.071 | 9.209 | 24/02/16 | 21/07/16 |
| Los Córdobas | Guaimaro Abajo | -76.364 | 8.861 | 9/03/16 | 3/08/16 |
| Montelíbano | Pica Pica Nuevo | -75.68 | 8.03 | 18/03/16 | 18/08/16 |
| TierraTierralta | Nueva Unión | -76.179 | 8.046 | 12/05/16 | 8/09/16 |
| Valencia | San Rafael | -76.246 | 8.16 | 25/05/16 | 25/08/16 |
| Puerto Libertador | San Juan | -75.727 | 7.734 | 2/06/16 | 14/09/16 |
