# Supplementary material for: Diversity patterns, Leishmania DNA detection, and bloodmeal identification of Phlebotominae sand flies in villages in northern Colombia
Source: PLoS One. 2018 Jan 10;13(1):e0190686. doi: 10.1371/journal.pone.0190686 (PMC5761875; doi:10.1371/journal.pone.0190686)
Supplement: S2 Table — (DOC) [file pone.0190686.s002.doc]

| **Municipality** | **Village** | **Total screened** | **Individuals per pool** | **Sandfly species** | **Conventional PCR** | **High Resolution Melting** | **Sequencing** |
| --- | --- | --- | --- | --- | --- | --- | --- |
| Lorica | Mata de Caña | 16 | 1 | *P. rangeliana* | + | Undetermined | No sequence |
| Lorica | Mata de caña |  | 1 | *L. gomezi* | + | *Leishmania panamensis* | *Leishmania panamensis* |
| Lorica | Mata de caña |  | 1 | *L. gomezi* | + | *Leishmania panamensis* | *Leishmania panamensis* |
| Lorica | Mata de caña |  | 1 | *P. rangeliana* | + | Undetermined | No sequence |
| Sahagún | Villa Lucía | 450 | 6 | *P. evansi* | + | Undetermined | No sequence |
| Sahagún | Villa Lucía |  | 7 | *P. evansi* | + | *Leishmania infantum* | *Leishmania infantum* |
| Sahagún | Villa Lucía |  | 7 | *P. evansi* | + | No melting curve | No sequence |
| Sahagún | Villa Lucía |  | 6 | *P. evansi* | + | No melting curve | No sequence |
| Sahagún | Villa Lucía |  | 11 | *P. evansi* | + | No melting curve | No sequence |
| Sahagún | Villa Lucía |  | 21 | *P. evansi* | + | No melting curve | No sequence |
| Planeta Rica | Punta Verde | 32 | 7 | *P. evansi* | + | No melting curve | No sequence |
| San Andres de Sotavento | Nueva Unión | 3120 | 9 pools (10 individuals) | *P. evansi* | + | *Leishmania infantum* | *Leishmania infantum* |
| San Andres de Sotavento | Nueva Unión |  | 4 pools (20 individuals) | *P. evansi* | + | *Leishmania infantum* | *Leishmania infantum* |
| Moñitos | Bellacohita | 655 | 20 | *P. evansi* | + | Undetermined | No sequence |
| Moñitos | Bellacohita |  | 7 | *L. gomezi* | + | *Leishmania panamensis* | *Leishmania panamensis* |
| Los Córdobas | Guaimaro Abajo | 405 | 2 pool (20 individuals) 1 pool (6 individuals) | *L. gomezi* | + | *Leishmania panamensis* | *Leishmania panamensis* |
| Montelíbano | Pica Pica Nuevo | 187 | 11 | *M. cayennensis* | + | *Leishmania panamensis* | *Leishmania panamensis* |
| Valencia | San Rafael | 214 | 16 | *P. panamensis* | + | *Leishmania panamensis* | *Leishmania panamensis* |
| San Andres de Sotavento | Hoja Ancha | 3514 | 20 | *P. evansi* | + | Undetermined | No sequence |
| San Andres de Sotavento | Hoja Ancha |  | 20 | *P. evansi* | + | No melting curve | No sequence |
| San Andres de Sotavento | Hoja Ancha |  | 20 | *P. evansi* | + | Undetermined | No sequence |
| San Andres de Sotavento | Hoja Ancha |  | 20 | *P. evansi* | + | *Leishmania infantum* | *Leishmania infantum* |
| San Andres de Sotavento | Hoja Ancha |  | 18 | *P. evansi* | + | *Leishmania infantum* | *Leishmania infantum* |
